# Silent predators: Revealing the parasites of Himalayan musk deer (*Moschus leucogaster*) in Manaslu Conservation Area, Nepal

**DOI:** 10.1016/j.ijppaw.2025.101119

**Published:** 2025-07-22

**Authors:** Bishnu Achhami, Shila Gurung, Sujan Deshar, Sapana Khaiju, Lekha Kumari Thapa, Sabita Gurung

**Affiliations:** a Small Mammals Conservation and Research Foundation, Kathmandu, 44600, Nepal; b Institute of Forestry, Pokhara Campus, Tribhuvan University, Pokhara, 33700, Nepal; c Himalayan Wolves Project, Salenstein, 8268, Switzerland; d Siddhartha Gautam Buddha Campus, Lumbini, Butwal, 32907, Nepal

**Keywords:** Gastrointestinal parasite, Fecal analysis, Strongyloides, Wildlife health, Endangered population, Conservation

## Abstract

Parasites pose a significant threat to wildlife, yet their impact remains largely understudied, with limited research conducted on the parasites of wild animals. This study provides the first quantitative analysis of gastrointestinal parasites in the endangered Himalayan musk deer (*Moschus leucogaster*), while also documenting a wider variety of parasites within the Manaslu Conservation Area (MCA), Nepal. Non-invasive sampling of 52 fresh fecal pellets from Kaltal (n = 28) and Mugumba (n = 24) revealed a high prevalence (94.2 %) of gastrointestinal parasites including two nematodes (*Pneumocaulus* sp. and Strongyle) and one parasitic protist (*Eimeria* sp.). *Pneumocaulus* sp. was the most abundant parasite, present in 100 % of Kaltal and 87.5 % of Mugumba samples. Strongyle was absent in Kaltal but had a lower prevalence (12.5 %) in Mugumba. Co-infection was identified in 25 % of samples, which could potentially threaten the health of musk deer. Statistical analysis with Firth's logistic regression indicated that higher elevations were associated with a lower chance of strongyle being present (p = 0.0057). Slope, aspect, and distance from water or roads did not significantly affect the distribution of the parasites. Parasite communities showed moderate similarity between the sites (Bray-Curtis dissimilarity = 0.22; Jaccard similarity = 0.67), with no significant difference in prevalence between Mugumba and Kaltal. *Pneumocaulus* sp. was found widely over the elevation gradient, most typically at 3600–3700 m, while strongyle was confined to lower elevations below 3500 m. This baseline study demonstrates the substantial gastrointestinal parasite burden in Himalayan musk deer and underscores the need for conservation and health management efforts for this endangered species in the MCA. Holistic conservation methods, including habitat management, disease detection, and further studies, including a large-scale sampling, seasonal data collection, and molecular techniques, can significantly enhance our understanding of the intricate relationship among parasites, hosts, and their environment in the endangered species conservation.

## Highlights

• First report of parasite in endangered Himalayan musk deer in Manaslu, Nepal.
• 94.2 % GI parasite prevalence indicates critical health risk for musk deer populations.
• Non-invasive fecal sampling and Firth's regression address small-sample challenges.
• Elevation drives strongyle distribution, highlighting climate-parasite linkages.
• Need of habitat management and disease control for musk deer conservation.

## Graphical abstract

**Figure:**
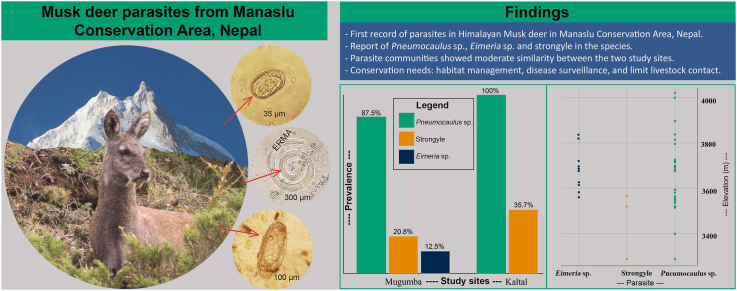

## 1 Introduction

Numerous parasite species inhabit the digestive tracts of ruminants; protozoan infections can especially be dangerous for infants and small animals (Jacobs et al., 2015) while parasitic diseases also pose serious threats to the health and productivity of ruminants (Hu et al., 2016). Due to parasites’ small size and limited visibility, their role in the functioning of ecosystems has been under-researched and underestimated, despite their frequently minimal direct impact on energy and material flows (Thomas et al., 2005). Parasites can provide insight into the impacts of climate change, urbanization, land use changes, and other variables on the health of humans and animals as well as the environment (Thompson and Polley, 2014).

Parasites are one of the major threats to wild animals, seriously affecting host reproduction and having the ability to decrease host density and even wipe out host populations (Roelke-Parker et al., 1996; Hudson et al., 1998; Ebert et al., 2000). Threatened mammal populations have been subjected to numerous known extinctions and near-extinctions due to disease (Berger, 1990), with smaller populations more at risk of extinction than larger ones (Figueiredo et al., 2016). Worldwide, the parasites of wild animals are among the least studied and researched subjects, with the parasites of small-sized mammals also being particularly understudied (Gómez and Nichols, 2013; Han et al., 2015; Brown et al., 2025). The inability to accurately diagnose infections in live animals has made large-scale wildlife surveillance difficult; however, molecular techniques now enable non-invasive sampling, where effective information collection will require global collaboration between ecologists, disease specialists, and conservation authorities (Thompson et al., 2010).

Musk deer are small-sized mammals, with seven species occurring in the Himalayan region of Asia. Of these, six species are listed as endangered (EN) and one as the vulnerable (VU) on the IUCN Red List (IUCN, 2024). Recent genetic research has confirmed two species of musk deer in Nepal: the Himalayan musk deer (*Moschus leucogaster*) and the Kashmir musk deer (*Moschus cupreus*), with the Manang and Kaski lineages in central Nepal identified as Himalayan musk deer (Singh et al., 2019). The male musk deer 's preputial gland secretes musk, a valuable substance that finds applications in perfumery and medicine (Green, 1986; Shrestha, 1998; Wang et al., 2020). Due to this musk deer face several threats, including poaching for musk, habitat loss (Yang et al., 2003; Homes, 2004), climate change (Jiang et al., 2020; Singh et al., 2020) and parasitic diseases (Yong-q, 2014; Gao et al., 2021; Wang et al., 2022).

The parasites of musk deer populations have not been extensively studied, although there has been some research on the parasites of captive musk deer (Song et al., 2018; Chen et al., 2022, Gao et al., 2021; Cui et al., 2022; Wang et al., 2022). Due to the limited budget for parasitic studies in Nepal, the parasites of wild animals are among the least studied subjects, with only one study (Achhami et al., 2016) focusing on the parasites of musk deer. Effective disease control is essential for the health and well-being of musk deer, since they are vulnerable to parasitic infections as a result of stress, environmental contamination, and conditions in captivity (Hu et al., 2018). The direct impact of parasitic diseases on musk deer population remains unclear due to the lack of information. In Nepal, parasitic infections may contribute to population declines of musk deer. This study aimed to identify gastrointestinal parasites in musk deer from the Manaslu Conservation Area, Gorkha, Nepal, establishing a baseline for future research; assess the influence of landscape features on the prevalence of parasites; evaluate the potential health impact of parasites on musk deer; and provide actionable recommendations for conservation.

## 2 Materials and methods

### 2.1 Study area

The Manaslu Conservation Area (MCA) located between 28.6°N and28.9°N latitude and 84.6°E and 85.1°E longitude is in the northern part of Nepal's Gandaki Province, within the Gorkha district. Established in 1998, it covers an area of 1663 square kilometers. It includes seven wards of the Tsum Nubri Rural Municipality: Samagaon, Lho, Kaltal, Bihi, Chumchet, Chhekampar, and Sirdibas. MCA is divided into three regions based on its ethnic diversity and its natural setting. The northeastern section, which includes the two wards of Chumchet and Chhekampar, is called Tsum Valley, while the middle section is known as Kutang. The region of the Budi Gandaki valley, west of Namrung, is known as Nubri Valley and is the northwest portion of the MCA that covers the Samagaun, Lho, and Kaltal wards. The altitude in MCA ranges from 600 m to the top of Mt. Manaslu (8163 m), the eighth-highest mountain in the world.

A rainy season runs from June to September and a dry season from October to May in the MCA. MCA is considered one of the global hotspots for biodiversity due to its location in the eastern Himalaya (Myers et al., 2000). In the MCA, five biological zones have been found (Shrestha, 2008). These zones include 19 different types of forests with roughly 2000 plant species (Rana, 2001), 39 animals, 201 birds, 3 reptiles, and 11 butterflies spread throughout 11 different forest types.

MCA is home to a variety of wildlife, including Himalayan tahr, Himalayan serow, Woolly hare, Himalayan marmot, Red fox, Jackal, Brown bear, Blue sheep, Snow leopards, and Musk deer.

**Fig. 1:**
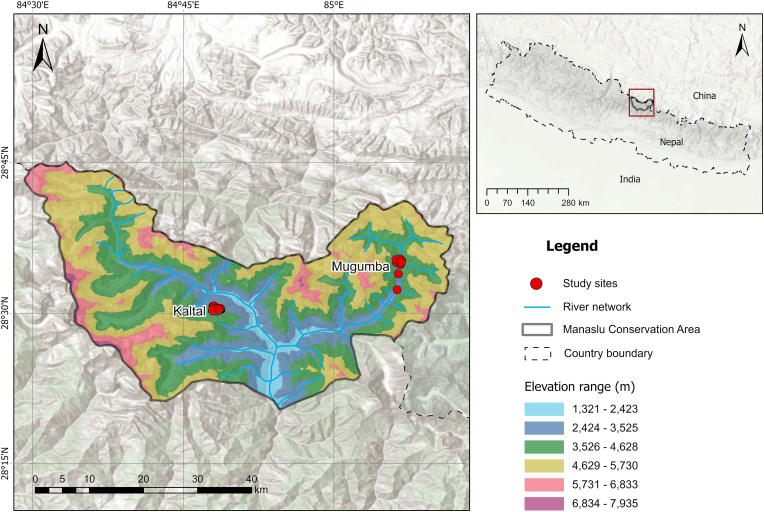
Map of study sites in Manaslu Conservation Area, Nepal.

Kaltal of Numbri Valley and Mugumba of Tsum Valley were the study areas of Manaslu Conservation Area (Fig. 1). The Kaltal area is located at the northwest side of MCA, and the Mugumba area is at the northeast side, where the aerial distance between these two places is 30 km.

### 2.2 Transect method and sample collection

A random transect survey was conducted in the habitat of Himalayan musk deer within the Manaslu Conservation Area. Transects were established in two areas (Kaltal and Mugumba), each transect measuring 150 m in length and 20 m in width (10 m on each side). The transect survey was conducted in the Kaltal area in December 2018 and in the Mugumba area in May–June 2019. The survey was carried out by moving slowly and quietly along the transect line, scanning both sides for the presence of latrine sites and fresh pellets of musk deer.

To ensure a representative sample, we collected one sample from each transect line, where available, based on the assumption of a latrine from one musk deer per transect. Samples were collected through non-invasive techniques, which include collecting fresh pellet samples directly from the ground using a sterile wooden spatula to avoid contamination. A total of 52 pellet samples of musk deer were collected, where 28 pellet samples were collected from the Kaltal and 24 pellet samples were collected from the Mugumba.

About 20 g of collected samples were placed in a sterile 50 mL polypropylene (PP) container with a polyethylene leak-proof screw cap. Then 70 % ethanol was placed in a 3:1 ratio to fully immerse the sample in ethanol to preserve and maintain its integrity until it reaches the lab. All samples were labeled with details such as location, date, and time of collection.

### 2.3 Lab process

In the lab, the pellet sample was first examined visually for any signs of parasites, such as worms or cysts. The concentration technique was used to identify gastrointestinal parasites of musk deer from pellets. First, about 3 g of pellets were ground into a fine piece using a mortar and pestle. A beaker was filled with fine pieces of pellets, to which 20 mL of distilled water were added. The mixture was then sieved through mesh screens to ensure a uniform particle size and to discard any undigested food materials. Then the sieved solution was poured into a 15 mL centrifuge tube, and the tube was centrifuged for 5 min at 2500 revolutions per minute (rpm). Again, the tube was centrifuged after the water was changed to a saturated flotation solution, zinc sulfate. After centrifugation, the centrifuge tube was placed in a test tube rack, and then more saturated zinc sulfate solution was added to the tube to form a reverse meniscus. This caused heavier fecal debris to settle at the bottom of the centrifuge tube while lighter parasitic particles rose to the top, forming a concentrated layer in the flotation solution. The cover slip was placed on the top of the centrifuge tube for about 5 min before being removed, placed on a slide, and examined under the microscope for identification of the parasites.

After examining the flotation section, the saturated zinc sulfate solution was carefully removed from the centrifuge tube. The sediment content was then poured into the watch glass, and the mixture was gently mixed. To get ready for the second slide, one drop of the mixture was taken, and Lugol's iodine wet mount solution was used to stain the mixture, which was then viewed beginning at 4x, then moving to 10x and 40x objective lens under a microscope to look for eggs, larvae, and trophozoites of parasites, and the color, shape, and size of the eggs and larvae were used to identify them (modified process of Zajac et al., 2021). Given the limited funds, laboratory equipment, and the resources available for parasitological studies on wild animals in Nepal, morphological identification is the only feasible method for identifying parasites.

Based on the morphological characteristics of the larvae and the available literature, we initially considered *Muellerius* sp. as a potential identification, due to features such as the nematode L1 larval morphology and the habitat being sympatric with livestock like sheep. However, after reviewing the recent short note on Musk deer parasites (Loginova and Maharjan, 2025) and analyzing the distinctive tail characteristics—including the prominent tail spike and longer dorsal spine compared to *Muellerius* sp., the L1 larval stage was identified as *Pneumocaulus* sp.

### 2.4 Statistical analysis

Statistical analyses were performed using R software (version 4.4.2). Descriptive statistics were calculated for all variables. We had the presence and absence of three parasites (*Eimeria* sp., Strongyle and *Pneumocaulus* sp.) as response variables. Landscape features (elevation, slope, aspect, proximity to water source and road) were used as predictor variables. We used QGIS 3.14 to prepare the study area map and quantify landscape features for analysis in R. Fisher's exact test was used to determine the relationship between the study sites (Mugumba and Kaltal) and the prevalence of gastrointestinal parasites. The binary result of parasite presence (1 = presence, 0 = absence) served as the response variable. We rescaled the covariates to ensure that all predictors were on a similar scale and checked collinearity among the covariates. The association between the prevalence and landscape features was assessed by univariable Firth's logistic regression analysis using the R package “*logistf*”. Firth's logistic regression is used to deal with small sample sizes and separation issues in binary logistic regression (D'Angelo and Ran, 2025). This method uses a penalized maximum likelihood approach to reduce small-sample bias in sparse data with separation issues. A difference was considered significant with a p-value ≤0.05. Because our dataset was small and had sparse observations, we used the *logistf.control()* function to increase the number of iterations for estimating the profile likelihood and calculating confidence limits. By raising the (*maxit*) value, we ensured the algorithm had enough steps to better optimize the penalized likelihood in Firth's logistic regression analysis, improving accuracy and reliability of the results (Heinze et al., 2013).

## 3 Results

**Fig. 2:**
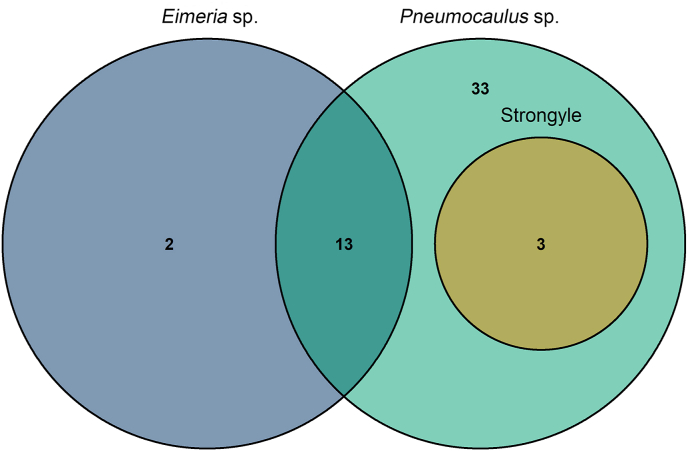
Venn diagram showing overlap of parasite presence in the pellets of Himalayan musk deer.

The overall prevalence of gastrointestinal parasites in the pellets of Himalayan musk deer was 94.2 % (49 out of 52 samples). Whereas the prevalence of parasites in the pellets from the Mugumba and Kaltal areas was 87.5 % (21 out of 24 samples) and 100 % (28 out of 28 samples), respectively. Two types of nematodes; *Pneumocaulus* sp. and strongyle, and one parasitic protist; *Eimeria* sp., were found in the pellets. Thirteen samples showed multiple parasitic infections of *Eimeria* sp. and *Pneumocaulus* sp., while 3 samples with strongyle and *Pneumocaulus* sp. (Fig. 2).

**Fig. 3:**
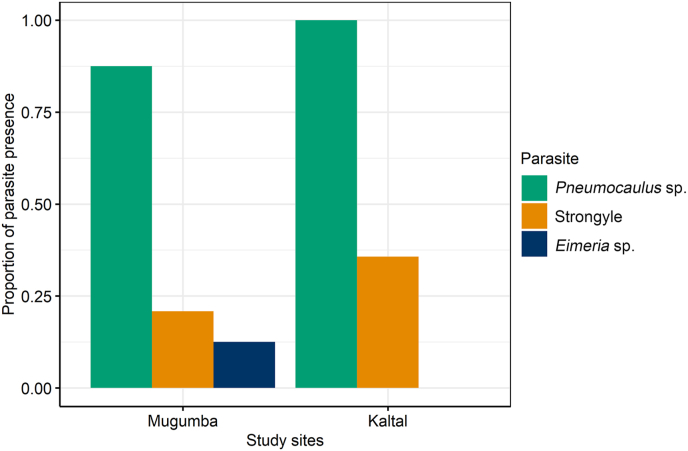
Prevalence of parasites from two study sites.

In the Mugumba area, the prevalence of *Pneumocaulus* sp., *Eimeria* sp., and strongyle parasites was 87.5 %, 20.8 %, and 12.5 %, respectively. In contrast, in the Kaltal area, the prevalence of *Pneumocaulus* sp. and *Eimeria* sp. was 100 % and 35.7 %, respectively, with an absence of the strongyle parasite (Fig. 3).

Fisher's exact test indicated that the association between the two study sites (Mugumba and Kaltal) and the presence of gastrointestinal parasites was not statistically significant (p-value >0.05 for all tests). Specifically, the p-values for *Pneumocaulus* sp., strongyle, and *Eimeria* sp. parasites were 0.0916, 0.0916, and 0.3582, respectively. A Bray-Curtis dissimilarity value of 0.22 showed that the parasite communities in Mugumba and Kaltal had highly similar compositions in terms of abundance. Meanwhile the Jaccard similarity value of 0.67 indicated that 67 % of the parasite species were shared between the two locations. This reflected a moderate overlap, with one-third of the parasite species found only in one of the two locations.

**Fig. 4:**
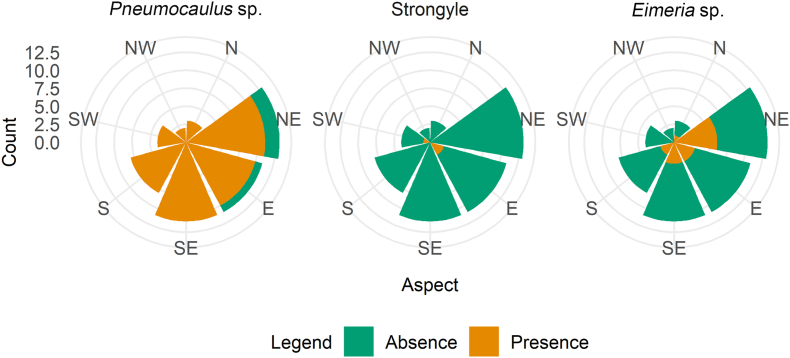
Rose diagram showing relation between parasite presence and aspects.

The presence of three parasites varied by aspect. *Pneumocaulus* sp. parasite was distributed across different aspects and most prevalent in the southeast (SE), east (E) and northeast (NE). Strongyle was mainly found in the east (E). *Eimeria* sp. presence was slightly higher in the northeast (NE) (Fig. 4).

**Fig. 5:**
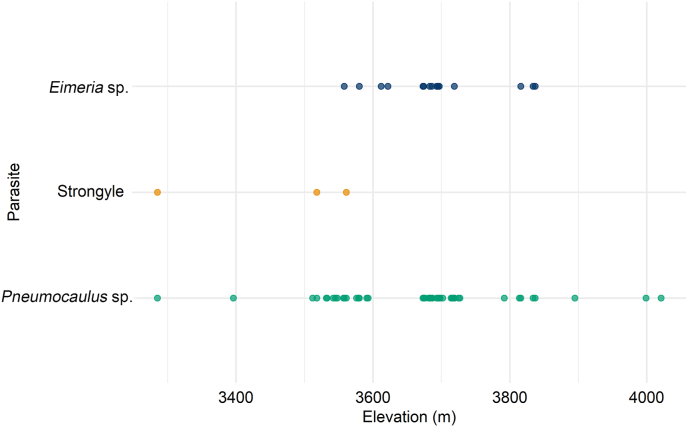
Scatter plot showing elevational distribution of parasites.

*Eimeria* sp. was primarily found at higher elevations between 3600 and 3800 m, while strongyle was restricted to lower altitudes below 3500 m. *Pneumocaulus* sp. was present across the entire elevational gradient. However, it was highly concentrated between 3600 and 3700 m (Fig. 5).

**Fig. 6:**
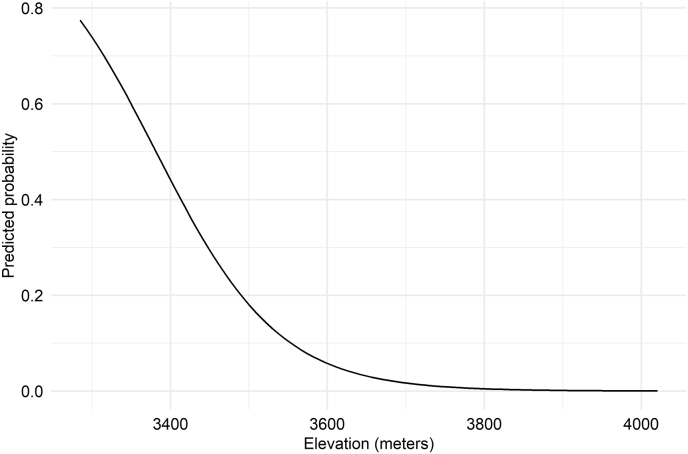
Predicted probability of strongyle prevalence across elevations.

The Firth logistic regression analysis revealed a significant negative association between strongyle presence and elevation (coefficient = −1.6735; p = 0.0057; Table 1), with higher elevations linked to a reduced likelihood of strongyle occurrence (Fig. 6). However, the other predictors, including slope (coefficient = −0.0923; p = 0.8689), aspect (coefficient = 0.1783; p = 0.7328), distance to water (coefficient = −0.5900; p = 0.3805), and distance to road (coefficient = −0.5068; p = 0.4798), did not show statistically significant effects on parasite presence. For *Eimeria* sp. and *Pneumocaulus* sp., none of the predictors showed statistically significant effects on parasite presence.

**Table 1:** Result of univariate Firth logistic regression analysis comparing the influence of landscape variables on parasite prevalence.

Model	Coefficients	SE	95 % CI		Chi-square	*p*
			lower	upper		
*Pneumocaulus* sp. (±)						
Elevation	0.6044	0.5076	−0.5494	1.7273	1.0705	0.3008
Slope	−0.4448	0.5393	−1.7088	0.6720	0.5854	0.4442
Aspect	1.0925	0.8383	−0.4196	3.5537	1.6182	0.2033
Distance to water	0.3110	0.6046	−0.7341	2.2757	0.2739	0.6007
Distance to road	6.9122	3.7943	−0.1019	14.4334	3.4306	0.0640
Strongyle (±)						
Elevation	−1.6735	0.6850	−3.6864	−0.4647	7.6541	0.0057
Slope	−0.0923	0.5155	−1.2041	1.0490	0.0273	0.8689
Aspect	0.1783	0.4726	−1.0707	1.0861	0.1165	0.7328
Distance to water	−0.5900	0.7216	−3.7924	0.5783	0.7692	0.3805
Distance to road	−0.5068	0.7472	−5.1141	0.6383	0.4994	0.4798
*Eimeria* sp. (±)						
Elevation	0.2872	0.2992	−0.2968	0.9180	0.9246	0.3363
Slope	−0.2220	0.2993	−0.8335	0.3657	0.5492	0.4587
Aspect	−0.1044	0.3015	−0.7584	0.4685	0.1203	0.7287
Distance to water	0.4116	0.2893	−0.1587	0.9943	2.0200	0.1552
Distance to road	0.2129	0.2811	−0.3637	0.7672	0.5604	0.4541

## 4 Discussion

This study aimed to assess gastrointestinal parasite prevalence and their relationship with landscape features in Himalayan musk deer in the Manaslu Conservation Area, a critical habitat for this endangered species in Nepal. The analysis revealed high prevalence of gastrointestinal parasites in the pellet samples, indicating a significant health concern for the populations of Himalayan musk deer within the Manaslu region. Both Mugumba and Kaltal sites showed high parasite prevalence, with no statistically significant difference between these two sites in terms of the presence or absence of gastrointestinal parasites. The high parasite prevalence indicated a potential vulnerability of musk deer to parasite-related diseases, which could negatively impact their conservation and overall health. However, geographic locations alone might not be a strong determinant of the prevalence of gastrointestinal parasites. The parasites might be able to survive in certain areas due to several local environmental factors or certain characteristics of the host animals (such as immunity or behavior) (Barron et al., 2015; Turner et al., 2021). Future research incorporating a larger sample size from diverse geographical regions is essential to comprehensively examine the potential factors, both facilitating and limiting, that influences the prevalence of parasites in musk deer.

The majority of fecal samples in this study exhibited multiple parasite infections, predominantly involving the parasitic protist *Eimeria* sp. and the nematode *Pneumocaulus* sp. As parasitic protists and helminths are essential members of parasite infracommunity (Pedersen and Fenton, 2007) collectively capable of infecting a single host. However, in some cases, one parasite prevents another from entering, growing, or reproducing inside the host (Shen et al., 2019). These parasites may then compete for the host's resources changing immune response, which can worsen health problems in musk deer or reduce their reproductive capacity (Budischak et al., 2015), ultimately affecting their survival. Considering the limited sample size in this study, future research exploring the behavioral and physiological impacts of multiple parasitic infections over a wider area could help improve our understanding of how gastrointestinal polyparasitism influence the ecology and conservation challenges of the musk deer.

In this study, *Pneumocaulus* sp. had the highest prevalence among parasites, followed by *Eimeria* sp. and strongyle. This might be because of the complex life cycles of the *Pneumocaulus* sp. that involve intermediate hosts (snails or slugs), which could enhance transmission opportunities (Saltini et al., 2023). Strongyle and *Eimeria* sp. on the other hand need specific habitats, hosts, or climates to exist which might limit their opportunity to transmit easily (Sun et al., 2018; Sweeny et al., 2022).

The presence of parasites was not uniformly distributed across different aspects, indicating that specific aspects may provide more favorable conditions for parasite survival and reproduction (Titcomb et al., 2022; Scott, 2023), as strongyle and *Eimeria* sp. were present in particular aspects, while *Pneumocaulus* sp. was present in all aspects. Understanding the underlying factors that influence parasite presence in different aspects could be crucial for parasite control and management strategies (Sweeny et al., 2022). *Pneumocaulus* sp. was also found across the widest range of elevations, indicating a high tolerance to varying environmental conditions across the elevations, while strongyle was found at lower elevations. Firth logistic regression further confirmed a significant negative association between elevation and strongyle prevalence in Himalayan musk deer, suggesting lower elevation below 3500 m might be more favorable for its prevalence. These patterns highlight elevation as a potential limiting factor for parasites distributions, supporting the theory that parasite diversity declines with increasing elevation (Moreno-Rueda, 2021). The finding reflected the possible combined influence of elevational climatic gradients on host-parasite relationships since factors such as temperature, humidity, vegetation, and host species distribution, all vary with elevation and could influence parasite survival and transmission (Scott, 2023; Hou et al., 2024; Khanal et al., 2024). Study by Zamora-Vilchis et al. (2012) also suggested strong association of parasite prevalence with regional climate conditions, especially annual temperature, besides altitude and physiological state of hosts (Santos and Ebert, 2022).

Globally, parasites have been studied in several musk deer species, including the Forest musk deer (*Moschus berezovskii*) (Hu et al., 2018; Song et al., 2018; Xu et al., 2018; Gao et al., 2021; Ren et al., 2021; Chen et al., 2022), the Alpine musk deer (*Moschus chrysogaster*) (Cui et al., 2022; Wang et al., 2022), the Siberian musk deer (*Moschus moschiferus*) (Kuznetsov et al., 2014; Kuznetsov et al., 2022; Loginova and Maharjan, 2025). However, no prior studies have investigated parasites in the Himalayan musk deer (*Moschus leucogaster*) in Manaslu Conservation Area, Nepal making this the first study on parasitic infections in this species in that area and filling a significant knowledge gap in musk deer parasitology. Additionally, this study emphasizes how environmental factors influence parasite distribution in this species.

Although non-invasive fecal collection is ethical and practicable for endangered species, it has limitations: it only identifies parasites shed in feces, potentially missing those in other organs and missing the total diversity of parasites affecting the host. Our findings suggest the need for larger-scale research, seasonally tracked studies, and additional methodologies to increase the accuracy of future research.

## 5 Conclusion

**This study fills an important gap in knowledge, being the first to target gastrointestinal parasites, while also documenting a broader spectrum of parasites in the endangered Himalayan musk deer within Nepal's Manaslu Conservation Area.** The findings provided insight into the relationship between parasite prevalence- *Pneumocaulus* sp., strongyle, and *Eimeria* sp.*—*and predictor landscape variables. Musk deer are already under threat from habitat loss, poaching, and climate change and face additional risks from parasitic infections. The results highlight the urgent need for comprehensive conservation measures. Conservation of this species and the ecological integrity of the Manaslu Conservation Area must be combined with habitat management, disease surveillance, and reduced livestock-wildlife contact to minimize parasite transmission.

## CRediT authorship contribution statement

**Bishnu Achhami:** Writing – review & editing, Writing – original draft, Visualization, Validation, Methodology, Investigation, Funding acquisition, Formal analysis, Data curation, Conceptualization. **Shila Gurung:** Writing – review & editing, Writing – original draft, Validation, Data curation. **Sujan Deshar:** Writing – review & editing, Writing – original draft, Data curation, Investigation. **Sapana Khaiju:** Investigation, Writing – original draft, Writing – review & editing. **Lekha Kumari Thapa:** Writing – original draft, Writing – review & editing, Investigation. **Sabita Gurung:** Writing – review & editing, Writing – original draft, Visualization, Validation, Supervision, Formal analysis, Data curation.

## Declaration of generative AI and AI-assisted technologies in the writing process

During the preparation of this work the authors used ChatGPT and QuillBot free version in order to improve the grammar and readability of the manuscript. After using this tool/service, the authors reviewed and edited the content as needed and takes full responsibility for the content of the publication.

## Funding

This work was supported by The 10.13039/100007463Rufford Foundation, Tottenham, London, in 2018 with the application ID: 24193-1.

## Declaration of competing interest

The authors declare the following financial interests/personal relationships which may be considered as potential competing interests: Bishnu Achhami reports financial support was provided by 10.13039/100007463The Rufford Foundation. If there are other authors, they declare that they have no known competing financial interests or personal relationships that could have appeared to influence the work reported in this paper.

## References

[bib1] Achhami B., Sharma H.P., Bam A.B. (2016). Gastro intestinal parasites of musk deer (*Moschus chrysogaster* Hodgson, 1839) in Langtang national park, Nepal. J. Inst. Sci. Technol..

[bib2] Barron D., Gervasi S., Pruitt J., Martin L. (2015). Behavioral competence: how host behaviors can interact to influence parasite transmission risk. Curr. Opin. Behav. Sci..

[bib3] Berger Joel (1990). Persistence of different‐sized populations: an empirical assessment of rapid extinctions in bighorn sheep. Conserv. Biol..

[bib4] Brown T.M., Dunn A.M., Quinnell R.J., Clarke E., Cunningham A.A., Goodman S.J. (2025). An interdisciplinary approach to improving conservation outcomes for parasites. Conserv. Biol..

[bib5] Budischak S.A., Sakamoto K., Megow L.C., Cummings K.R., Urban J.F., Ezenwa V.O. (2015). Resource limitation alters the consequences of co-infection for both hosts and parasites. Int. J. Parasitol..

[bib6] Chen S., Meng W., Shi X., Chai Y., Zhou Z., Liu H., Zhong Z., Fu H., Cao S., Ma X., Shen L., Deng L., Peng G. (2022). Occurrence, genetic diversity and zoonotic potential of *Blastocystis* sp. in forest musk deer (*Moschus berezovskii*) in Southwest China. Parasite.

[bib7] Cui Z., Wang Q., Huang X., Bai J., Zhu B., Wang B., Guo X., Qi M., Li J. (2022). Multilocus genotyping of *Giardia duodenalis* in alpine musk deer (*Moschus chrysogaster*) in China. Front. Cell. Infect. Microbiol..

[bib8] D'Angelo G., Ran D. (2025). Tutorial on Firth's logistic regression models for biomarkers in preclinical space. Pharm. Stat..

[bib9] Ebert D., Lipsitch M., Mangin K.L. (2000). The effect of parasites on host population density and extinction: experimental epidemiology with *Daphnia* and six microparasites. Am. Nat..

[bib10] Figueiredo A., Oliveira L., Madeira de Carvalho L., Fonseca C., Torres R.T. (2016). Parasite species of the endangered Iberian wolf (*Canis lupus signatus*) and a sympatric widespread carnivore. Int. J. Parasitol. Parasites Wildl..

[bib11] Gao Y., Duszynski D.W., Yuan F., Hu D., Zhang D. (2021). *Eimeria sp.* parasites in the endangered Forest Musk Deer (*Moschus berezovskii*) in China, with the description of six new species of *Eimeria* (Apicomplexa: Eimeriidae). Parasite.

[bib13] Gómez A., Nichols E. (2013). Neglected wild life: parasitic biodiversity as a conservation target. Int. J. Parasitol. Parasites Wildl..

[bib14] Green M.J.B. (1986). The distribution, status and conservation of the Himalayan musk deer *Moschus chrysogaster*. Biol. Conserv..

[bib15] Han B.A., Schmidt J.P., Bowden S.E., Drake J.M. (2015). Rodent reservoirs of future zoonotic diseases. Proc. Natl. Acad. Sci..

[bib16] Heinze G., Ploner M., Dunkler D., Southworth H. (2013). Firth's bias reduced logistic regression. R Package Versionn.

[bib18] Homes V. (2004). Traffic Europe.

[bib19] Hou L., Liang Y., Wang C., Zhou Z. (2024). Mineral protection explains the elevational variation of temperature sensitivity of soil carbon decomposition in the Eastern Himalaya. Appl. Soil Ecol..

[bib20] Hudson P.J., Dobson A.P., Newborn D. (1998). Prevention of population cycles by parasite removal. Science.

[bib21] Hu X.L., Liu G., Wang W.X., Zhou R., Liu S.Q., Li L.H., Hu D.F. (2016). Methods of preservation and flotation for the detection of nematode eggs and *Eimeria* sp. oocysts in faeces of the forest musk deer. J. Helminthol..

[bib22] Hu X.-L., Liu G., Wei Y.-T., Wang Y.-H., Zhang T.-X., Yang S., Hu D.-F., Liu S.-Q. (2018). Regional and seasonal effects on the gastrointestinal parasitism of captive forest musk deer. Acta Trop..

[bib23] IUCN (2024). The IUCN red list of threatened species. https://www.iucnredlist.org.

[bib24] Jacobs D., Fox M., Gibbons L., Hermosilla C. (2015).

[bib25] Jiang F., Zhang J., Gao H., Cai Z., Zhou X., Li S., Zhang T. (2020). Musk deer (*Moschus* spp.) face redistribution to higher elevations and latitudes under climate change in China. Sci. Total Environ..

[bib26] Khanal S., Nolan R.H., Medlyn B.E., Boer M.M. (2024). Disentangling contributions of allometry, species composition and structure to high aboveground biomass density of high-elevation forests. Ecol. Manag..

[bib61] Kuznetsov D.N., Seryodkin I.V., Maksimova D.A (2022). Nematodes of the digestive tract of musk deer in Primorsky Krai. Theory Practice Parasitic Dis. Control.

[bib27] Kuznetsov D.N., Seryodkin I.V., Maksimova D.A., Khrustalev A.V. (2014). On the helminthofauna of *Moschus moschiferus*. Achiev. Life Sci..

[bib29] Loginova O.A., Maharjan B. (2025).

[bib31] Moreno-Rueda G. (2021). Elevational patterns of blowfly parasitism in two hole nesting avian species. Diversity (Basel).

[bib32] Myers N., Mittermeier R.A., Mittermeier C.G., Da Fonseca G.A.B., Kent J. (2000). Biodiversity hotspots for conservation priorities. Nature.

[bib33] Pedersen A.B., Fenton A. (2007). Emphasizing the ecology in parasite community ecology. Trends Ecol. Evol..

[bib34] Rana A.S.J.B. (2001).

[bib59] Ren Z., Yu D., Zhao W., Luo Y., Cheng J., Wang Y., Yang Z., Yao X., Yang W., Wu X., Li Y. (2021). Investigation and molecular identification of Eimeria sp. sampled from captive forest musk deer. PeerJ.

[bib35] Roelke-Parker M.E., Munson L., Packer C., Kock R., Cleaveland S., Carpenter M., O'Brien S.J., Pospischil A., Hofmann-Lehmann R., Lutz H., Mwamengele G.L.M., Mgasa M.N., Machange G.A., Summers B.A., Appel M.J.G. (1996). A canine distemper virus epidemic in Serengeti lions (*Panthera leo*). Nature.

[bib36] Saltini M., Vasconcelos P., Rueffler C. (2023). Complex life cycles drive community assembly through immigration and adaptive diversification. Ecol. Lett..

[bib37] Santos J.L., Ebert D. (2022). The effects of temperature and host-parasite interactions on parasite persistence in a planktonic crustacean. J. Freshw. Ecol..

[bib38] Scott M.E. (2023). Helminth-host-environment interactions: looking down from the tip of the iceberg. J. Helminthol..

[bib39] Shen S.-S., Qu X.-Y., Zhang W.-Z., Li J., Lv Z.-Y. (2019). Infection against infection: parasite antagonism against parasites, viruses and bacteria. Infect. Dis. Poverty.

[bib40] Shrestha M.N. (1998). Animal welfare in the musk deer. Appl. Anim. Behav. Sci..

[bib41] Shrestha T.B. (2008). Classification of Nepalese forests and their distribution in protected areas. Initiation.

[bib42] Singh P.B., Khatiwada J.R., Saud P., Jiang Z. (2019). mtDNA analysis confirms the endangered Kashmir musk deer extends its range to Nepal. Sci. Rep..

[bib43] Singh P.B., Mainali K., Jiang Z., Thapa A., Subedi N., Awan M.N., Ilyas O., Luitel H., Zhou Z., Hu H. (2020). Projected distribution and climate refugia of endangered Kashmir musk deer *Moschus cupreus* in greater Himalaya, South Asia. Sci. Rep..

[bib44] Song Y., Li W., Liu H., Zhong Z., Luo Y., Wei Y., Fu W., Ren Z., Zhou Z., Deng L., Cheng J., Peng G. (2018). First report of *Giardia duodenalis* and *Enterocytozoon bieneusi* in forest musk deer (*Moschus berezovskii*) in China. Parasites Vectors.

[bib45] Sun P., Wronski T., Bariyanga J.D., Apio A. (2018). Gastro-intestinal parasite infections of Ankole cattle in an unhealthy landscape: an assessment of ecological predictors. Vet. Parasitol..

[bib46] Sweeny A.R., Corripio-Miyar Y., Bal X., Hayward A.D., Pilkington J.G., McNeilly T.N., Nussey D.H., Kenyon F. (2022). Longitudinal dynamics of co-infecting gastrointestinal parasites in a wild sheep population. Parasitology.

[bib47] Thomas F., Renaud F., Guegan J.-F. (2005). Parasitism and Ecosystems.

[bib48] Thompson R.C.A., Lymbery A.J., Smith A. (2010). Parasites, emerging disease and wildlife conservation. Int. J. Parasitol..

[bib49] Thompson R.C.A., Polley L. (2014). Parasitology and one health. Int. J. Parasitol. Parasites Wildl..

[bib50] Titcomb G.C., Pansu J., Hutchinson M.C., Tombak K.J., Hansen C.B., Baker C.C.M., Kartzinel T.R., Young H.S., Pringle R.M. (2022). Large-herbivore nemabiomes: patterns of parasite diversity and sharing. Proc. Biol. Sci..

[bib51] Turner W.C., Kamath P.L., van Heerden H., Huang Y.-H., Barandongo Z.R., Bruce S.A., Kausrud K. (2021). The roles of environmental variation and parasite survival in virulence–transmission relationships. R. Soc. Open Sci..

[bib52] Wang J., Xing H., Qin X., Ren Q., Yang J., Li L. (2020). Pharmacological effects and mechanisms of muscone. J. Ethnopharmacol..

[bib53] Wang Q., Liu X., Li Y., Xin L., Zhou X., Yu F., Zhao A., Qi M. (2022). Genetic diversity of *Blastocystis* subtypes in the Alpine musk deer (*Moschus chrysogaster*) in Gansu province, northwestern China. J. Eukaryot. Microbiol..

[bib60] Xu J., Lin H., Chen J., Cai Y., Fu W., Wang H., Gu X., Lai W., Peng X., Yang G. (2018). Molecular characterization of Moniezia sichuanensis in captive musk deer (Moschus berezovskii). J. Helminthol..

[bib55] Yang Q., Meng X., Xia L., Feng Z. (2003). Conservation status and causes of decline of musk deer (*Moschus* spp.) in China. Biol. Conserv..

[bib56] Yong-q W. (2014). The current status and disease occurrence of captive forest musk deer (*Moschus berezovskii*) in Shaanxi Province. Chin.J. Ecol..

[bib57] Zajac A., Conboy G.A., Little S.E., Reichard 1973- M.V., Parasitologists A.A. of V. (2021).

[bib58] Zamora-Vilchis I., Williams S.E., Johnson C.N. (2012). Environmental temperature affects prevalence of blood parasites of birds on an elevation gradient: implications for disease in a warming climate. PLoS One.

